# Vaccine-induced immune thrombotic thrombocytopenia in Tunisia following AstraZeneca COVID-19 vaccination: a case report

**DOI:** 10.11604/pamj.2025.52.56.48175

**Published:** 2025-10-02

**Authors:** Mohamed Ksentini, Mariam Ammar, Nour El Houda Ben Ali, Ahmed Hakim, Zouheir Sahnoun, Khaled Zeghal, Lobna Ben Mahmoud

**Affiliations:** 1 Regional Pharmacovigilance Service, Sfax, Tunisia; 2 Department of Pharmacology of Sfax, Faculty of Medicine of Sfax, Sfax University, Sfax, Tunisia; 3 Laboratory of Research of Pharmacology and Toxicology (LR19ES10), University of Sfax, Sfax, Tunisia

**Keywords:** AstraZeneca COVID-19 vaccination, vaccine-induced thrombotic thrombocytopenia, cerebral infarcts, anti-platelet factor, case report

## Abstract

Vaccines against SARS-CoV-2 were rapidly developed to combat the COVID-19 pandemic. Rare cases of Vaccine-Induced Immune Thrombotic Thrombocytopenia (VITT) have been reported after the AstraZeneca COVID-19 vaccination. We present a case of a 51-year-old healthy man who developed fever, unconsciousness, thrombocytopenia, multiple thromboses, and macrophage activation syndrome one week after receiving the first dose of the AstraZeneca COVID-19 vaccine. Initial anti-PF4 antibody tests were negative, but subsequent ELISA confirmed their presence. Despite plasma exchange and steroid therapy, the patient developed neurological deficits, gastrointestinal hemorrhage, multiple cerebral infarcts, and died. This case illustrates the risk of fatal vaccine-induced immune thrombotic thrombocytopenia after an AstraZeneca COVID-19 vaccination and emphasizes the need for early recognition and specialized testing, including PF4 ELISA, to improve patient outcomes. Reporting such cases raises awareness, supports timely diagnosis, and guides appropriate management while reinforcing the importance of vaccination.

## Introduction

Vaccine-induced immune thrombotic thrombocytopenia (VITT) is a rare but severe complication occurring after adenoviral vector COVID-19 vaccination, particularly following AstraZeneca COVID-19 vaccination (ChAdOx1 nCoV-19, Vaxzevria®) [1-3]. This syndrome combines immune-mediated thrombocytopenia with thrombotic events at atypical sites, including cerebral venous sinuses and splanchnic veins. It is associated with the presence of anti-platelet factor 4 (PF4) antibodies in the absence of prior heparin exposure, mimicking the mechanism of heparin-induced thrombocytopenia [4,5]. Symptoms usually develop within 5-30 days after AstraZeneca COVID-19 vaccination and can include severe headache, visual disturbances, abdominal pain, shortness of breath, limb swelling, nausea, or vomiting [2,5]. Laboratory findings often show thrombocytopenia and markedly elevated D-dimer levels, and imaging may reveal thrombosis in unusual locations [4,6]. Although VITT remains extremely rare, awareness is critical because early recognition and appropriate management can prevent progression to life-threatening complications [7-9]. We report a fatal case of VITT in a 51-year-old man occurring one week after the first dose of the AstraZeneca COVID-19 vaccination. The case was reported to the Regional Pharmacovigilance Service of Sfax, Tunisia. Causality assessment was performed using the WHO AEFI (Adverse Event Following Immunization) criteria [10].

## Patient and observation

**Patient information:** a 51-year-old healthy man with no reported medical history received the first dose of the AstraZeneca COVID-19 vaccine. He had no history of cardiovascular disease, venous thrombosis, or prior COVID-19 infection.

**Timeline of current episode:** the patient received the first dose of the AstraZeneca COVID-19 vaccine on day 0. On day 3, he developed a persistent fever despite taking paracetamol for five days. On day 7, he was found unconscious with a body temperature of 41°C and was transported immediately to the intensive care unit. At admission, he was intubated, mechanically ventilated, and an oral anticoagulant, apixaban, was started.

**Clinical findings:** on day 10, the patient developed edema in his lower limbs. Laboratory tests showed thrombocytopenia with a platelet count of 48x10^9^/L (baseline 159x10^9^/L), elevated D-dimer levels of 4500 ng/mL (reference <500 ng/mL), and macrophage activation syndrome confirmed on bone marrow puncture. Doppler ultrasound revealed bilateral deep humoral vein thrombosis and superficial left posterior tibial thrombosis.

**Diagnostic assessment:** initial anti-platelet factor 4 (PF4) antibody testing by indirect particle gel immunodiffusion assay was negative. The case was reported to the regional pharmacovigilance service on day 12. On day 13, ELISA confirmed the presence of anti-PF4 IgG antibodies. Noninvasive cerebral imaging using CT and MRI initially showed no cerebral thrombosis. Other causes of thrombocytopenia and thrombosis, including infection, cardiomyopathy, and prior COVID-19 infection, were excluded.

**Diagnosis:** based on the WHO AEFI, the patient was diagnosed with Vaccine-Induced Immune Thrombotic Thrombocytopenia (VITT) following AstraZeneca COVID-19 vaccination. This was based on thrombocytopenia, multiple thrombotic events, macrophage activation syndrome, and positive anti-PF4 antibodies despite no prior exposure to heparin.

**Therapeutic interventions:** the patient underwent three plasma exchange sessions combined with steroid therapy. Apixaban was administered as an anticoagulant. On day 47, he developed swallowing disturbances and motor deficits, with electromyography (EMG) showing peripheral polyradiculoneuritis, prompting another plasma exchange session.

**Figure 1:**
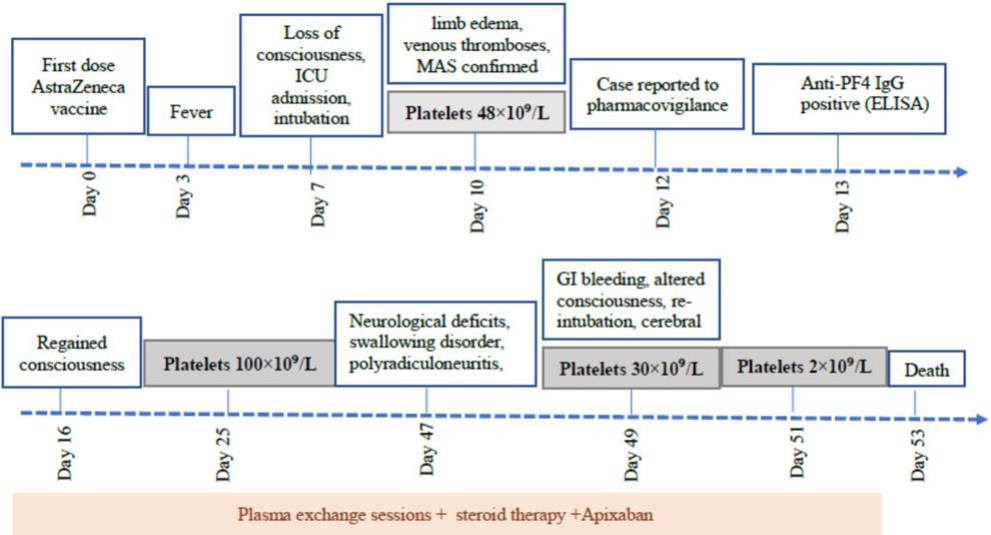
clinical course of the patient with vaccine-induced immune thrombotic thrombocytopenia after AstraZeneca COVID-19 vaccination

**Follow-up and outcome of interventions:** on day 16, the patient regained consciousness two days after sedation was discontinued. By day 25, platelet count improved to 100x10^9^/L. On day 49, he developed acute gastrointestinal bleeding and altered consciousness, was re-intubated and mechanically ventilated, and multiple cerebral infarcts were detected on a computed tomography (CT) brain scan. Platelet count dropped from 30x10^9^/L to 2x10^9^/L by day 51. The patient died on day 53. The clinical course of the patient is summarized in Figure 1.

**Patient perspective:** this was not applicable since the patient was critically ill and deceased.

**Informed consent:** the case was reported to the regional pharmacovigilance service as part of routine pharmacovigilance. The case report is presented anonymously, and all identifying information has been removed to protect patient confidentiality.

## Discussion

Vaccine-induced immune thrombotic thrombocytopenia (VITT) is a rare but severe adverse effect following adenovirus vector COVID-19 vaccination. The risk has been most consistently associated with the AstraZeneca COVID-19 vaccine (ChAdOx1 nCoV-19). Large case series have reported an estimated incidence of approximately 1 in 100,000 vaccinated individuals [1,2]. A systematic review including post-marketing data showed that the incidence varies by age and sex, with the highest rates observed in younger adults, particularly women under 50 years [3]. According to the EMA Pharmacovigilance Risk Assessment Committee (PRAC), by June 2021, nearly 479 suspected cases had been identified in the EU/EEA, of which around 100 were life-threatening [3]. Although the clinical presentation is often severe, these events remain extremely rare compared to the overall number of doses administered. Current evidence emphasizes that the benefits of the AstraZeneca COVID-19 vaccination in preventing severe COVID-19 far outweigh the risks of VITT.

Symptoms of VITT typically appear 5-20 days after vaccination. Patients often present with severe headache, visual disturbances, abdominal pain, shortness of breath, or limb swelling. This clinical profile prompted the EMA to issue a “Dear Doctor Letter” alerting physicians to the need for early recognition [5]. The hallmark of VITT is the presence of anti-platelet factor 4 (PF4) antibodies, detected even in the absence of prior heparin exposure. This mimics the mechanism of heparin-induced thrombocytopenia but occurs independently of heparin [4,7]. Thrombocytopenia combined with thrombosis at unusual sites, such as splanchnic or cerebral veins, supports the diagnosis. Laboratory confirmation relies on enzyme-linked immunosorbent assays (ELISA) specific for PF4 antibodies, as rapid immunoassays are less sensitive in this context [4]. Post-mortem confirmation is challenging because antibody testing is not routinely performed during forensic autopsies, limiting diagnostic certainty in fatal cases [9].

In our patient, the temporal relationship with vaccination, absence of alternative causes, and detection of anti-PF4 antibodies by ELISA confirmed the diagnosis of fatal VITT. The pathophysiology likely involves immune-mediated activation against PF4, potentially triggered by vaccine components forming neoantigens or by interactions of the spike protein with endothelial cell heparan sulfate proteoglycans, which promote platelet activation and thrombus formation [7,8].

Vaccine-induced immune thrombotic thrombocytopenia can cause severe thrombotic complications with high mortality, particularly in cerebral and splanchnic vein thrombosis. Reported fatality rates range from 30% to over 50% [1-3]. Early recognition and rapid treatment are critical. Heparin should be avoided because the syndrome mimics heparin-induced thrombocytopenia. Recommended alternatives include direct oral anticoagulants or argatroban [2]. Intravenous immunoglobulin (IVIG) has demonstrated benefit by blocking Fcγ receptor-mediated platelet activation, while corticosteroids and plasma exchange are reserved for refractory cases [1,2]. Despite these strategies, outcomes remain poor in a significant proportion of patients. Delayed diagnosis, extent of thrombosis, and severe thrombocytopenia are key predictors of fatality [3]. In our case, the disease progressed rapidly and led to death despite recognition and testing, consistent with the unfavorable outcomes reported in the literature.

## Conclusion

We report the first fatal case of VITT following an AstraZeneca COVID-19 vaccination in Tunisia. Early recognition of symptoms, prompt laboratory confirmation of anti-PF4 antibodies, rapid initiation of appropriate non-heparin anticoagulation, and timely reporting to pharmacovigilance authorities are critical to improve outcomes. Clinicians should remain vigilant for VITT in patients presenting with thrombosis and thrombocytopenia within 5-20 days after adenoviral vector vaccination, as delayed diagnosis can be fatal.
